# Heparin: Past, Present, and Future

**DOI:** 10.3390/ph9030038

**Published:** 2016-07-04

**Authors:** Eziafa I. Oduah, Robert J. Linhardt, Susan T. Sharfstein

**Affiliations:** 1 SUNY Polytechnic Institute, Albany, NY 12203, USA; eoduah@bhs1.org or eoduah@albany.edu; 2 Department of Medicine, Berkshire Medical Center, Pittsfield, MA 01201, USA; 3 Rensselaer Polytechnic Insitute, Troy, NY 12180, USA; linhar@rpi.edu

**Keywords:** heparin, heparan sulfate, heparin-like molecules, bioengineering, UFH, low molecular weight heparin, anti-inflammatory, antitumor, Chinese hamster ovary cells

## Abstract

Heparin, the most widely used anticoagulant drug in the world today, remains an animal-derived product with the attendant risks of adulteration and contamination. A contamination crisis in 2007–2008 increased the impetus to provide non-animal-derived sources of heparin, produced under cGMP conditions. In addition, recent studies suggest that heparin may have significant antineoplastic activity, separate and distinct from its anticoagulant activity, while other studies indicate a role for heparin in treating inflammation, infertility, and infectious disease. A variety of strategies have been proposed to produce a bioengineered heparin. In this review, we discuss several of these strategies including microbial production, mammalian cell production, and chemoenzymatic modification. We also propose strategies for creating “designer” heparins and heparan-sulfates with various biochemical and physiological properties.

## 1. History and Background

Heparin is the oldest anticoagulant used in clinical medicine. Paradoxically, heparin was discovered by Mclean in 1916 in an attempt to isolate a thromboplastic agent [1]. Heparin is a naturally occurring polysaccharide belonging to the family of glycosaminoglycans (GAG) ubiquitously present in mast cells. Further work eventually led to its inception into clinical use in 1935. Since then, heparin has been studied for various applications and modifications.

**Figure 1:**
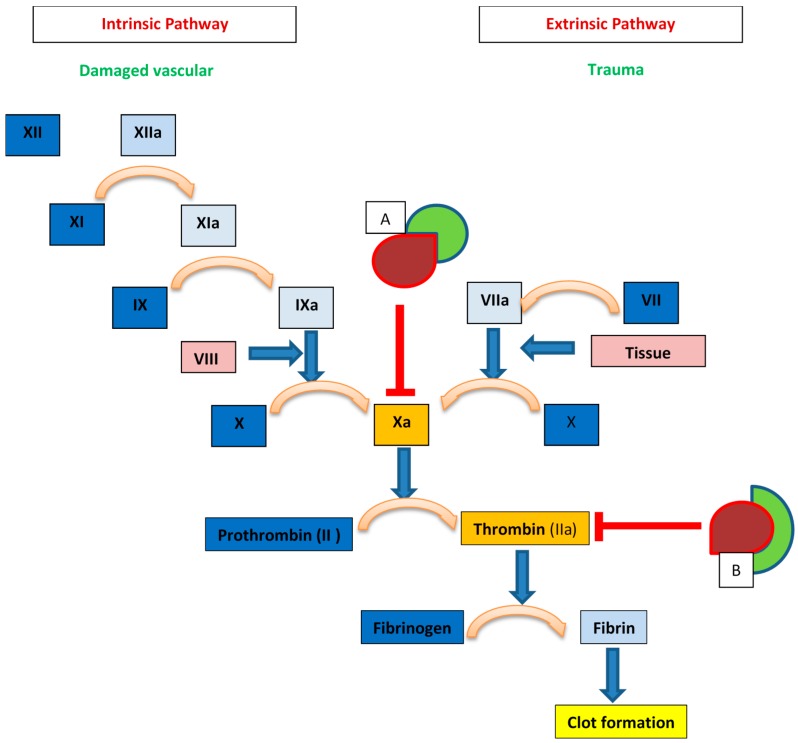
Heparin mechanisms within the coagulation cascade. Box A: AT (red) bound with heparin fragments (green) of any length within the unique pentasaccharide sequence can inhibit factor Xa. Box B: AT (red) bound with heparin (green) with chain length >17 disaccharide units can inhibit thrombin (Factor IIa).

Unfractionated heparin (UFH) is the least processed form of the natural GAG produced via purification from animal tissue, most commonly porcine intestine. Its introduction into clinical medicine was a monumental advance in the 1930s due to the paucity of clinically available anticoagulant alternatives. Decades of research has provided additional insight into its structure and mechanism of anticoagulant activity, albeit, our understanding of heparin is still incomplete. It is now known that heparin exerts its anticoagulant properties indirectly by binding with antithrombin III (AT) and facilitating the subsequent inhibitory effect of AT on thrombin and activated factor X (factor Xa) [2,3]. Only UFH containing at least 18 saccharide sequences can influence the action of AT on thrombin; however, UFH fragments of any length containing a unique pentasaccharide sequence can inhibit the action of factor Xa [4,5] (Figure 1).

Due to the heterogeneity of its structure, the bioactivity and physiological action of UFH is broad and unpredictable. Some heparin chains bind to other plasma proteins, with side effects including adverse consequences on bone metabolism in the form of osteoporosis, heparin induced thrombocytopenia (HIT), and unpredictable anticoagulation requiring continuous monitoring [6]. Further research and development resulted in the introduction of low molecular weight heparins (LMWH) in the late 1970s to early 1980s, in an attempt to produce a more predictable activity profile.

LMWH, such as enoxaparin, dalteparin and tinzaparin, are prepared via controlled chemical or enzymatic cleavage of UFH in a depolymerization reaction [7]. This controlled process yields fragments of lower molecular weight and more predictable action than UFH. The result is a better adverse reaction profile than the UFH, less requirements for monitoring, higher bioavailability, and the potential for outpatient administration [8,9]. Therefore, LMWH became the standard of care in place of UFH except in certain scenarios such as renal failure and acute coronary syndromes where UFH is still preferred due to ease of hepatic clearance and better reversibility with protamine sulfate.

Ultra-low molecular weight heparins (ULMWH) were developed in the early 2000s through synthetic chemical processes. The rationale was to produce agents with an even better side effect profile while promoting similar or better anticoagulant properties consequent to a higher anti-factor Xa to anti-thrombin activity ratio [10,11]. Although some ULMWH have found clinical use elsewhere in the world, they are not widely implemented in the US [12], due in part, to their higher cost, resulting in a low benefit-to-cost ratio.

## 2. Overview of Structure and Biosynthesis of Heparin

**Figure 2:**
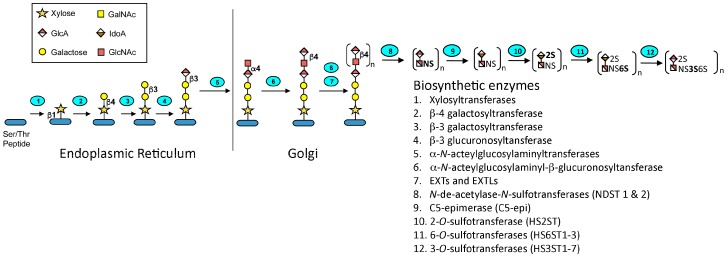
Heparin biosynthesis. The glycosaminoglycan-protein linkage region is first formed under the action of glycosyltransferases. The repeating disaccharide units are then elongated by GlcA and GlcNAc transferases. Chain modifications including *N*-deacetylation and *N*-sulfonation, *O*-sulfonations, and epimerization then occur under the actions of the specified enzymes. Monosaccharide symbols in this figure follow the SNFG (Symbol Nomenclature for Glycans) system [15]

Heparin is a highly sulfated and polydisperse GAG with molecular weights ranging between 5–40 kDa. It has a complex structure consisting of repeating disaccharide units consisting of uronic acid residues (l-iduronic (IdoA) or d-glucuronic acid (GlcA)) and *N*-acetyl-d-glucosamine [13]. The biosynthesis of heparin (Figure 2) occurs primarily in the endoplasmic reticulum and Golgi apparatus of mast cells. A tetrasaccharide linker is attached to a serine residue on a core protein, serglycin, and then the d-glucuronic acid (1→4) *N*-acetyl-d-glucosamine disaccharide units are added. Sulfonation of the disaccharides and epimerization of the glucuronate to iduronate is carried out by various enzymes in the biosynthetic pathway. There are a total of 12 enzymes involved in the pathway, which act in concert to produce the desired molecule. However, many of these enzymes have several isoforms, which may account for the heterogeneity of heparin and allows these enzymes to direct the biosynthesis of the related glycosaminoglycan, heparan sulfate. The degree of sulfation and localization of the sulfate residues determines the spectrum of activity of the product. Upon mast cell degranulation, peptidoglycan heparin is transformed to the GAG heparin through the action of proteases and β-endo glucuronidase [14].

## 3. Novel Approaches to Synthesis

The annual market for heparin is ~7 billion dollars [16]. Most of this heparin is currently obtained from animal tissue under less than ideal current good manufacturing practice (cGMP) conditions. A 2008 crisis of public health significance resulting in about 100 deaths in the United States alone, in addition to other adverse reactions, was attributed to contamination of heparin samples with oversulfated GAGs produced under such conditions [17]. Since then, several approaches to bioengineering of heparin have been under investigation. These include chemoenzymatic modification, microbial production, and mammalian cell production, as discussed below.

### 3.1. Mammalian Cell Production

Mammalian cell production of heparin has been attempted using Chinese hamster ovary (CHO) cells. CHO cells are currently one of the most commonly used mammalian cells to produce biologic pharmaceuticals due to their unique properties such as robustness, safety from potential viral contamination, and ease of manipulation. These cells are also considered favorable for the biologic engineering of heparin since they intrinsically express most of the enzymes implicated in the heparin biosynthetic pathway with the exception of two, HS3ST1 and NDST2 [18]. These two enzymes are essential for the anticoagulant properties of heparin as they are involved in the synthesis of the AT-binding pentasaccharide and *N*-sulfation of GlcNAc, respectively [19]. Furthermore, CHO cells naturally produce heparan sulfate (HS), a GAG similar to heparin, sharing the same biosynthetic pathway, but without the anticoagulant properties of heparin [20,21].

Initial efforts to produce heparin in CHO cells involved metabolic engineering of the two deficient enzymes into the cells. Analysis of the heparinoid secreted from selected stable engineered cell lines showed an increase in anticoagulant activity by approximately 100-fold compared to the nonengineered cell lines [22]. However, in comparison to pharmaceutical heparin, the anticoagulant activity was subpar. Structural analysis of the final product by reverse-phase ion-pairing ultra-performance liquid chromatography mass spectrometry (RPIP-UPLC-MS) showed increased sulfation and total GAG production in the engineered cells compared to the wild type [23]. However, there was a significant structural variation from pharmaceutical heparin; the engineered HS had an increase in *N*-sulfation, but not the subsequent 2-*O*-sulfation and 6-*O*-sulfation, which are required to create the trisulfation common in pharmaceutical heparin.

Furthermore, analysis showed that while the engineered NDST2 was primarily localized to the Golgi and endoplasmic reticulum, HS3ST1 was dispersed in the Golgi, endoplasmic reticulum, and cytoplasm. This observed mistargeting of HS3ST1 was proposed to be a culprit for the inferior anticoagulant property of engineered HS and led to further work targeting HS3ST1 to the Golgi. In this later work, the anti-factor Xa activity was increased, as was the overall anticoagulation activity. However, only a single type of AT-binding site was formed, as compared to multiple AT-binding sites in pharmaceutical heparin [24], again indicating that the engineered product was not yet equivalent to pharmaceutical heparin.

One other limitation identified for the biosynthesized heparinoid from CHO cells was low productivity compared with the quantities of recombinant proteins that are typically produced in engineered CHO cells. To address the low productivity, bioprocess optimization was performed in the hope of increasing productivity of the bioengineered heparinoid similar to what is seen in the production of biopharmaceutical proteins. Initial optimization was performed in fed-batch shake flasks where the integrated viable cell density increased approximately two-fold and the specific productivity increased approximately 70%, resulting in nearly three-fold increase in product titer. Transferring the process to a stirred-tank bioreactor increased the productivity further, yielding a final product concentration of ~90 μg/mL. Furthermore, increasing the sulfur availability in the feed by supplementing with cysteine increased the anticoagulant activity approximately two-fold in fed-batch shaker flask studies although no change was observed in disaccharide composition [25].

Together, these findings represent a significant milestone towards bioengineering of heparin using mammalian cells. It is likely that these findings will pave a way for further research, which may include multidisciplinary approaches to manipulation of cell lines, as well as identification of novel applications for the engineered heparinoid molecule. Another strategy with the potential for future application is the CRISPR (clustered regularly interspaced short palindromic repeats)/Cas9 technology. Since the Doudna and Charpentier labs developed the CRISPR/Cas 9 gene-editing tool in 2012, this technology has enjoyed widespread application in the scientific community. This technology has the advantage of ability to upregulate or downregulate the expression of the enzymes involved in the biosynthetic pathway through an efficient genome editing process. It has been proposed for possible application towards metabolic engineering of heparin [26]. By controlling the biosynthetic pathway, this approach can be used to modify the anticoagulant or growth factor binding profiles of the engineered heparin in CHO cells. For example, knocking out HS3ST5 would likely remove all anticoagulant activity, while knocking out NDST1 would reduce binding to fibroblast growth factor. Moreover, this technology has been demonstrated in microbial systems using *E. coli* to metabolically engineer naringenin, a heterologous plant flavonoid. Therefore, it might be applied to current systems to improve the quality of the heparin product and possibly increasing the productivity of the engineered cell lines.

### 3.2. Chemoenzymatic Approaches

Successful chemical synthesis of heparin and related substances is manifest in the drug fondaparinux, a synthetic analog of the pentasaccharide sequence for AT binding required for Factor Xa inhibition. This drug, under the brand name of Arixtra, is approved for the treatment of deep-vein thrombosis. It is also approved for the treatment of HIT. However, the high cost of this medication has limited its clinical use to situations where less costly alternatives are contraindicated or poorly tolerated. The high cost is attributed, in part, to the multiple tedious steps involved in chemical synthesis, as well as the high cost of the resources required, in addition to overall low yield [27]. Recently, chemoenzymatic synthesis has been proposed as an alternative approach to producing either structurally defined LMWH or ULMWH or a more polydisperse product using microbial-derived heparosan as a precursor.

Chemoenzymatic synthesis relies on the action of polymerases for the formation and elongation of a backbone, and further modification under the action of recombinant heparin biosynthetic enzymes, including sulfotransferases and C5 epimerase [28]. This method has been demonstrated with varying levels of success by several authors. One group reported the synthesis of an ULMWH, a fondaparinux-like molecule, through a 12-step process of backbone elongation and chain modification steps. The anticoagulant profile of the synthesized molecule was similar to that of fondaparinux. This result suggested that targeted and controlled chemoenzymatic synthesis of heparin-like drugs can be feasibly undertaken. A more recent study expanded the feasibility of the chemoenzymatic approach in synthesizing a LMWH up to dodecsaccharide length utilizing the same approach. The results of the pharmacokinetic studies suggested a similar anticoagulant profile to other LMWH, potential for reversibility with protamine, and the possibility of renal clearance [29].

An alternative approach to chemoenzymatic synthesis of monodisperse heparinoids has been developed using the bacteria *E. coli* K5. *E. coli* K5 is a natural producer of the polysaccharide heparosan, an unsulfated “precursor” of the heparin and HS produced in eukaryotic cells. The initial study using this system was not favorable as it resulted in the formation of a “neoheparin” containing unnatural sequences with chemical similarities to the implicated contaminant of the 2008 health crisis [30]. However, this study paved the way for further studies using the same bacterial heparosan. Further work showcased a system whereby a heparosan backbone [31] was converted into a product more similar to pharmaceutical heparin through *N*-deacetylation and *N*-sulfation steps, followed by modification by recombinant C5 epimerase and OSTs [26].

Limitations of chemoenzymatic synthesis include substrate specificities of the enzymes, which may limit the variety of structures produced, and challenges in performing large-scale synthesis cost-effectively to meet the clinical need [28]. Strategies to overcome some of these limitations have been proposed including process control, incorporation of metabolic engineering, and culture optimization [26]. Some of these have been attempted such as the one-pot synthesis in which a heparosan precursor is mixed with all of the appropriate heparin biosynthetic enzymes simultaneously. This approach, which requires optimization of the relative enzyme concentrations, does offers the potential for scale up [32]; however, others are yet to see fruition.

## 4. Novel Applications of Heparin

Apart from its use as an anticoagulant, over the years there has been growing interest in the potential applications of heparin for other purposes. These applications range from anti-inflammatory and anti-tumor applications to prevention of infectious disease and use as nanocarriers for drug delivery.

### 4.1. Inflammatory and Allergic Disorders

Interest in the anti-inflammatory effects of heparin dates back several decades. Case reports of subjective improvement in patients with moderate to severe chronic, obstructive pulmonary disease led to a double-blind trial in the 1960s. This study suggested relief of bronchospasms and obstructing mucous secretions in patients that received intravenous heparin administration [33]. Following that preliminary study, several other animal and human studies reported similar findings in the treatment of bronchopulmonary disease [34]. In a study involving 24 asthma patients, the effect of treatment with enoxaparin, a LMWH, was evaluated. The authors reported an increase in the forced expiratory volume in one second (FEV1), which is an assessment of airway obstruction or bronchoconstriction. They also reported a decrease in the percentage of eosinophils and lymphocytes upon bronchoalveolar lavage, which corresponds to a reduction in inflammation [35]. Another study demonstrated attenuation of post-exercise bronchoconstriction in patients with exercise-induced asthma [36]. A recent systematic review suggested the role of heparin was to reduce the histamine or leukotriene-induced bronchial hyper-reactivity, without inhibiting the bronchoconstriction response [36]. This systematic review did not reveal any significant adverse events from the use of any form of heparin, except for an increase in partial thromboplastin time in one study [37].

Although the evidence appears to favor a role for heparin and related derivatives in the treatment of patients with asthma, the results for other inflammatory diseases have been equivocal. For example, findings for ulcerative colitis (UC), an inflammatory disorder of the bowel, have been controversial. One study in 2000, comparing corticosteroids with heparin for the treatment of UC, found that heparin was not effective as a mono therapy and was, in fact, associated with increased risk of bleeding [38]. However, another study found heparin to be effective as a monotherapy for the treatment of severe ulcerative colitis [39]. LMWH was effective when administered as an extended colon-release tablet. Findings of a recent review suggest that the beneficial effect of heparin and its derivatives may be related to its anticoagulant activity [37].

The mechanisms behind the anti-inflammatory effect of heparin have yet to be completely elucidated. Proposed mechanisms include binding of inflammatory cytokines and acute phase reactions by heparin, inhibition of adhesion molecules involved in the inflammation response, most importantly P-selectin and l-selectin [40], inhibition of NF-κB translocation from the cytoplasm to the nucleus [41], and upregulation of apoptosis via the TNF-α and NF-κB pathways [34].

### 4.2. Malignancies

The potential role of heparin in the management of malignancy has been under investigation in recent decades. Interest in the role of heparin was spurred by the observation that patients with malignancies who received concomitant treatment with heparin or its LMW derivatives for venous thromboembolism (VTE) had better outcomes than those who did not. The preclinical and clinical evidence for the antitumor properties of LMWH have been studied and reviewed extensively [42,43,44], suggesting that LMWH decreased mortality in cancer patients; however, a recent meta-analysis showed a controversial result reporting no survival benefit with the addition of LMWH [45]. The more recent FRAGMATIC trial comparing the addition of 24 weeks of prophylactic dose of the LMWH, dalteparin to standard therapy versus standard therapy alone in lung cancer patients showed that dalteparin did not improve overall survival in lung cancer patients [46]. It may be that the inconsistency in results is attributable to the use of different heparins, possessing different antimetastatic activity profiles. For example, tinzaparin has a higher selectin-inhibitory activity compared to dalteparin, which may confer a greater antimetastatic effect [47]. The mechanisms of the antineoplastic properties of heparin have been the subject of several studies. The antineoplastic activity appears to be unrelated to the anticoagulant properties. Proposed chemotherapeutic mechanisms include interference with cellular proliferation, release of tissue factor pathway inhibitor (TFPI) from vascular endothelium, anti-inflammatory properties, and inhibition of heparanase activity resulting in decreased tumor invasion and metastasis [48,49]. One of the most relevant mechanisms of heparin inhibition of hematogenous spread of malignant cells appears to be via inhibition of P-selectin mediated platelet adhesion to tumor cells [50] and l-selectin mediated leukocyte interaction with tumor cells [51].

Although VTE prophylaxis with anticoagulant therapy is indicated in hospitalized cancer patients and those receiving chemotherapy, the risks of bleeding with the use of heparin and its derivatives has precluded its implementation in the management of cancer patients in other therapeutic settings. In addition, studies have shown that the antitumor, anti-angiogenetic and anti-metastatic properties of heparin are unrelated to its anticoagulant effect [52,53]. Hence, non-anticoagulant species of heparin (NACH) were developed with the hope of decreasing the risks of bleeding while improving the antitumor profile. Several NACH variants have been developed, with varying degrees of antitumor vs. bleeding profiles [54]. One study showed an increased antimetastatic profile of a NACH compared to the LMWH tinzaparin [53]. Another NACH showed superiority to the LMWHs tinzaparin and enoxaparin in a mouse model of pancreatic cancer, with increased antitumor properties and reduction in bleeding time when compared to the LMWH controls [54,55]. In addition, there were no reported toxic effects in this study.

### 4.3. Infectious Diseases

The potential application of heparin derivatives in the field of infectious disease is less studied compared to inflammatory and oncologic conditions. However, they are also under investigation for potential use as antimicrobial agents due to their inhibitory effects on pathogen adhesion to cell surfaces. The pathogenesis of most infectious diseases involves adherence of microbes to cell surfaces by means of cell surface adhesion molecules in which heparan sulfate proteoglycans play a role [56]. Adhesion is usually followed by internalization of the organism into the cell, which, in turn, leads to other downstream effects of the infectious process on the cellular level. This process occurs for most bacterial and viral pathogens, although the downstream effector processes may differ [57,58]. Other proposed mechanisms for the use of HP/HS derivatives in this field include their potential use in the area of diagnostics, as well as application towards further understanding of multidrug resistant microbial organisms that employ capsular polysaccharides as part of their virulence factors [58]. Overall, more studies are required in this field for future applications.

### 4.4. Heparin Molecules as Nanocarriers for Drug Delivery

Due to the antitumor properties of heparin, there is a growing interest in the use of heparin for functionalization of nanocarriers for targeted drug delivery in cancer treatment. Functionalization is accomplished through the formation of covalent bonds with the nanoparticle core or through electrostatic interactions. The rationale for using heparin for this purpose stems from the effects discussed above, including its ubiquitous presence in cells, antitumor, anti-inflammatory, and anticoagulant properties. Furthermore, heparin confers novel properties to nanoparticles such as stealth, which enables nanoparticles to bypass clearance by the reticuloendothelial system [59], improved targeting of molecules to tumor cells with enhanced uptake and accumulation [60], and increased stability and solubility of gold nanoparticles [61]. The use of heparin for improved functionality of nanocarriers is the subject of an extensive review [62].

## 5. Structural Modifications of Heparin to Achieve Better Profile for Non-Anticoagulant Functions

Different modifications of heparin and HS molecules influence the spectrum of their anticoagulant, antitumor, and anti-inflammatory properties. For example, sulfation at the C3 glucosamine residue is a rare sulfation event; however, it is especially significant in several biologic activities of heparin and its derivatives. While it has been known that HS3ST-regulated sulfation is essential for the antithrombin activity of heparin, which impacts its anticoagulant properties, newer evidence has suggested a potential role for 3-*O*-sulfation in inhibiting fibroblast growth factors (FGF) involved in mitogenic and migratory functions of tumor cells [62]. Furthermore, the length of oligosaccharide chains may affect the activity of basic fibroblast growth factor (bFGF). According to the results of one study, dodecsaccharide and octasaccharide chain lengths of heparin and its derivatives had the potential to inhibit bFGF while those with 12 oligosaccharide units did not exhibit an inhibitory effect [63].

As alluded to earlier, the antitumor and antimetastastic properties of heparin are also closely related to its heparanase-inhibition properties. One study suggests that inhibition of heparanase activity was better achieved by heparin species containing 16 or more sugar units in addition to *N*- and *O*-sulfation. Oligosaccharides with higher sulfation levels exhibited higher heparanase inhibition activity [64]. In another study, increased heparanase-inhibitory activity was seen with removal of 2-*O*-sulfo and 3-*O*-sulfo groups and removal of its carboxyl groups by 50 and 18 percent respectively; moreover, heparanase activity was completely annihilated by removal of all three structures [47]. It has also been suggested that sulfation at the C6 glucosamine residue is important for potentiating the inhibitory effect of heparin on selectins, which in turn inhibits cell adhesion and migration [65].

A heparin derivative with enhanced antitumor/antiproliferative activity was reported in a study in which LMWH was chemically modified using butyric anhydride. The resultant activity spectrum of the butanyolated heparin in a lung cancer model included an increase in apoptotic index of tumor cells, increased inhibition of tumor cell proliferation, as well as decreased anticoagulation activity, both in vitro and in vivo. They reported no toxic effects of the modified heparin compared to the UFH; rather the bleeding side effect profile was reduced compared to UFH. However, structural analysis of the butanyolated heparin was not reported [66].

The available evidence provides a framework for further manipulation of heparin and HS to engineer derivatives with different spectra of activity and potential use as future therapeutics.

## 6. Future Perspectives and Directions

Heparin is a naturally occurring substance with several identified physiologic properties and potential for more diverse applications than currently employed. Currently, heparin (including UFH, LMWH, ULMWH) is most commonly used as an anticoagulant. The heparin-related health crisis in 2007–2008 called for better ways of manufacturing safe, anticoagulant heparin. In addition, there is a growing concern about a shortage of porcine heparin given the enormous number of animals that must be slaughtered in order to meet the current need. To this end, effort has been directed towards developing and improving techniques and approaches towards the synthesis of heparin as described above. To date, some degree of success has been achieved. However, more work is required in specific approaches including improving anticoagulant profiles of the engineered heparinoids, improved scalability for better yield, more preclinical and clinical trials to establish efficacy and safety profiles.

The specific structures responsible for the anticoagulant properties of heparin have been studied. Several chemical, chemoenzymatic, and metabolic engineering techniques for structural modification of heparin have also been developed over the years, again with varying degrees of success. These structures may be modified and/or used in concert to achieve desired structures for heparin geared towards specific purposes. Moreover, more studies will be required both at the preclinical and clinical stages to study the in vivo effects of these heparin- and HS-like molecules to determine their pharmacokinetic and safety profiles. Whether or not HP/HS-derived molecules will find clinical applications is yet to be determined; however, given the era of increased prevalence of certain types of oncologic malignancies, paucity of effective anti-inflammatory medications with narrow side effect profiles, as well as growing antibiotic resistance, there may be a window of opportunity for application in these fields as well.

In conclusion, within a century of its discovery, heparin has been successfully applied in several clinical scenarios and continues to be one the most commonly used anticoagulants today. Several advances have been made towards understanding the mechanisms of action and spectrum of its biological activities. New insight has also been gained into potential ways for bioengineering and synthesis of heparin and heparin-like molecules geared towards meeting the current need for safety and mitigating the concern for shortages in the current supply. In spite of these advances, there remain opportunities for improvement. Some of these include better understanding of the structure-function relationship of the compound, fine-tuning the loose ends of the synthesis and engineering process, potential diversification of applications to include the antitumor, anti-inflammatory and/or the area of infectious diseases. Surface modification of nanoparticles with heparin can also be used for diagnostic and therapeutic purposes in the field of oncology, although nanomedicine in general is an emerging field and more research into safety and efficacy are still required both at the preclinical and clinical levels.
